# What could evolve in the evolution of memory?

**DOI:** 10.1098/rstb.2024.0109

**Published:** 2025-06-26

**Authors:** Ellouise Leadbeater, Cecylia Watrobska

**Affiliations:** ^1^ Centre for Biodiversity and Environment Research, Department of Genetics, Evolution and Environment, University College London, London, UK; ^2^ Department of Biological Sciences, Royal Holloway University of London, Egham, UK; ^3^ Centre for Environmental Policy, Imperial College London, London, UK

**Keywords:** behaviour, memory, cognition, evolution

## Abstract

Over the past five decades, advances in neuroscience have set out the adaptive landscape of memory, illuminating semi-independent storage mechanisms, forgetting mechanisms and modifications to basic machinery that bring context specificity. Yet because much of neuroscience aims to understand how the human brain functions, rather than to explore taxonomic diversity, the implications for animal cognitive evolution remain underexplored. This perspective article examines the potential evolutionary diversity of animal memory from a mechanistic viewpoint. We argue that taking into account neurogenetic and neurophysiological mechanisms of memory could illuminate how the diversity of cognitive traits has been shaped by natural selection. By focussing on memory in insects, notably through the lens of associative processes, we target our discussion on potential variation in taxonomically general processes within one of the animal kingdom’s richest and most diverse animal groups. This exploration aims to broaden the discourse on memory evolution within the field of cognitive ecology towards an understanding of the many ways in which memory could be shaped by natural selection.

This article is part of the Theo Murphy meeting issue ‘Selection shapes diverse animal minds’.

## 1. Introduction

Memory is the fundamental building block that underlies many key processes of animal cognition. It optimizes behaviour, shapes sophisticated phenotypic plasticity and is present in some form in nearly all tested animal species, including molluscs, arthropods, annelids and nematodes [1–3]. What is more, the past five decades have seen transformative progress in understanding how truly multidimensional animal memory is. While it has long been understood that memories do not simply travel along a linear path from short- to long-term storage repositories, the field of neuroscience has now precisely disentangled multiple semi-independent memory phases, linked them to underlying neural structures, explored the mechanisms that connect them, and identified key genes that can suppress them [4–11]. We can now even see images of the physical form of different memories in real brains—the long-sought *engrams*, or unique networks that develop when a memory is formed, and whose subsequent reactivation re-awakens that memory [12]. The complexity of memory has never been more obvious, nor—thanks to the extraordinary array of tools now available to visualize and control different neurons—more tangible [13]

With rare exceptions (e.g. [14–18]), this progress in understanding the neurogenetic and neurophysiological basis for memory has yet to inform ideas about how natural selection might have moulded it differently across species, and why. For neuroscience, exploring diversity is not the key point; neuroscience uses animal models, but the aim is usually to understand what might happen in the human brain [19]. By contrast, for those interested in cognitive evolution, mechanisms are important but not an overriding focus because natural selection acts on behavioural phenotypes, rather than the neurons that produce them or the genes that control them. However, mechanisms are important in understanding cognitive evolution for two reasons. Firstly, they can uncover diversity that might not be immediately apparent through the behaviour of wild animals. For example, as we will discuss below, evidence that longer-term memory has different forms in insects has revealed interesting variation in the use of one form over another [16,20,21]. Secondly, mechanisms shape the adaptive landscape, determining what could evolve through small or low-cost tweaks to established machinery, and what would be a major innovatory leap. This is particularly relevant to questions over the independence of cognitive abilities across contexts or tasks that attract much debate in the field of cognitive ecology [22–25], which has historically drawn more heavily from psychology than neuroscience. Neural mechanisms may not be the focus of evolution, but they are certainly helpful in terms of understanding what could evolve and why.

In this brief perspective, we seek to motivate thinking about how some key aspects of memory, such as acquisition, short- and longer-term storage, and forgetting, *could* evolve in response to natural selection. We think these axes will be useful for characterizing diversity and relating it to ecological selection pressures, and in particular, we are interested in the evolution of ‘poor’ memory. When thinking about cognitive evolution, it is tempting to focus on those traits that involve exceptional feats of storage, accuracy or longevity. For example, the ability of food-storing bird species to remember hundreds of specific cache locations over a winter season is extraordinary, and clearly deserving of the research attention that it attracts [26,27]. However, to focus only on those outstanding, highly noticeable instances would be to ignore that *failing* to store experiences in long-term memory (LTM), or allowing them to be easily overwritten, is also a product of natural selection that needs to be explained [28]. For example, as we will discuss below, the process of forgetting is not simply an inescapable feature of natural decay processes, but also an active process that can be suppressed by downregulating particular target genes [11]. Just as the evolution of animal body allometry would not be well captured by a single axis from ‘smaller’ to ‘larger’, it does not make sense to characterize memory as ‘better’ or ‘worse’ as is sometimes the case within the cognitive ecology literature [20].

Although not exclusively, we focus on well-studied associative processes, and mostly on one taxonomic group: the insects. Most of the terrestrial animal species on the planet belong to this group [29,30]. Their ecological niches are diverse, as are their cognitive abilities, at least as far as we can see from the species that have been studied from a cognitive perspective, e.g [31–33]. However, it is the exceptionally rich history of neurobiological research into insect memory, particularly in the fruit fly *Drosophila melanogaster* [8,9,11], that sets them apart for our purposes (although several of the examples we discuss derive from the rodent literature). Our perspective is ecological, and we could not hope to capture the multidimensional integration of complex neural systems that underlie memory in depth: for this, we direct readers to recent reviews [9,12,14,34–36]. Instead, we aim to explore some of the fundamental dimensions of memory that might, or might not, be modifiable by natural selection, to generate ideas about whether and how each might evolve to meet specific ecological tasks.

## 2. What is memory?

Memory is classically defined as a process—the acquisition, storage and retrieval of information [37]—rather than an entity. However, the concept of a neural trace that underlies memory, constituting its physical form, dates from ancient Greece and was formalized in the mid-twentieth century as a network of interconnected cells that are simultaneously activated during an experience, inducing changes to the synapses that connect them [36,38]. Since then, evidence for neural correlates of memory has become rich and diverse, encompassing multiple model species [36,37,39,40]. Thanks to the development of tools that allow neuronal activity to be visualized and up/downregulated, it is now even possible to see the neural trace (the *engram*) that is formed on exposure to a stimulus or stimuli [41], to artificially reactivate the same trace to reinstate the memory [42] and to remove it to prevent recall forever [43]. Tracing of engram networks in rodents has uncovered their wide-ranging reach across the brain, providing evidence for sub-ensembles in different brain regions that are linked to produce the overall complex [12]. For example, fear conditioning is represented by multiple nodes across the thalamus, hippocampus and cortex and beyond [44]. Engrams can also incorporate multiple cell types, such as place cells and/or time cells, which encode specific locations and moments in time, respectively, and have been suggested to provide a potential physiological trace for components of episodic memory [45–47]. The potential of these approaches to evidence linkage between the processes underlying memory across different tasks, and to explore how re-networking of the same neural regions might allow for evolutionary change [48], is exciting.

**Figure 1.**
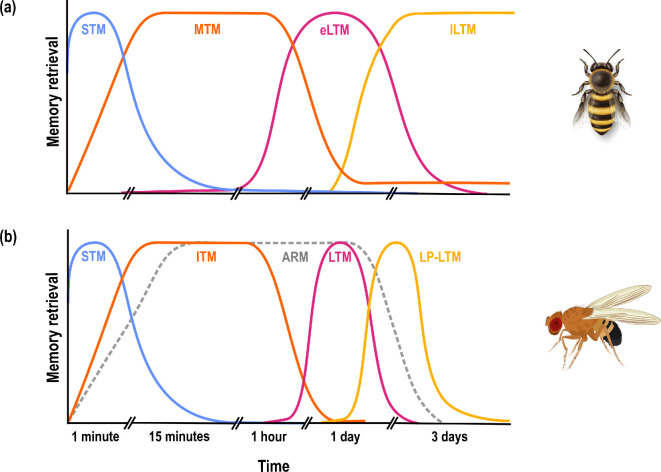
Models of memory phases in (a) honeybees (*Apis mellifera*; appetitive conditioning) and (b) fruit flies (*Drosophila melanogaster*; aversive conditioning). Memory is generally divided into short-term (STM), intermediate-term (ITM, also referred to as middle-term MTM) and long-term (LTM) memory phases, although the timings for each phase vary between species and protocols. Memory in honeybees is generally measured using appetitive conditioning protocols, and LTM is further divided into an early (eLTM) and a late (lLTM) phase, which are characterized by translation only, or transcription and translation, respectively. By contrast, memory in fruit flies is generally measured using aversive conditioning protocols. Flies can form a type of long-term memory (LTM) that is not dependent on protein synthesis (anaesthesia-resistant memory, ARM), and a late-phase long-term memory (LP-LTM) that is protein synthesis-dependent. Honeybee figure based on [14]; fly figure based on [8,9,36].

Alongside evidence that the neural traces of memories span multiple brain regions that might perform separate sub-tasks within a memory, there is also evidence that the different temporal categories of memory may take place in different neuronal populations [8]. Memory has long been understood as occurring in multiple different forms, each of which is characterized by its own specific physiology and temporal dynamics; for example, at least five different temporal phases exist in *D. melanogaster,* including short-term (STM) and intermediate-term memory (also known as middle-term memory), anaesthesia-resistant memory (ARM) and two forms of protein-synthesis dependent long-term memory (LTM and late phase-LTM) [9]. Moreover, while there are basic consistencies, the timing and basic physiology of these phases can differ across tasks (e.g. appetitive vs aversive conditioning [49]) and across species ([8,14,20]; figure 1a,b). The description of these forms as ‘phases’ can give the impression that memories progress linearly from one phase into another, an idea that does not capture the complex reality. For example*,* protein-synthesis dependent LTM formation begins during training, and takes place in parallel with STM formation, not as a sequel to it [5,7]. In *D. melanogaster,* STM and LTM each require different populations of neurons in the mushroom bodies [9,50,51], which are an integrative centre associated with memory formation. Furthermore, there is evidence to suggest that ARM and LTM are alternative longer-term storage repositories with different energetic demands [52], and that memories cannot be stored in both [4], as we discuss further below. Thus, the physical basis for memory is an enormously complex entity that varies in space and in time. However, what aspects of it could evolution act on? What should we be looking for, to see how variation might match the demands of different ecological or social niches?

## 3. Acquisition

**Figure 2.**
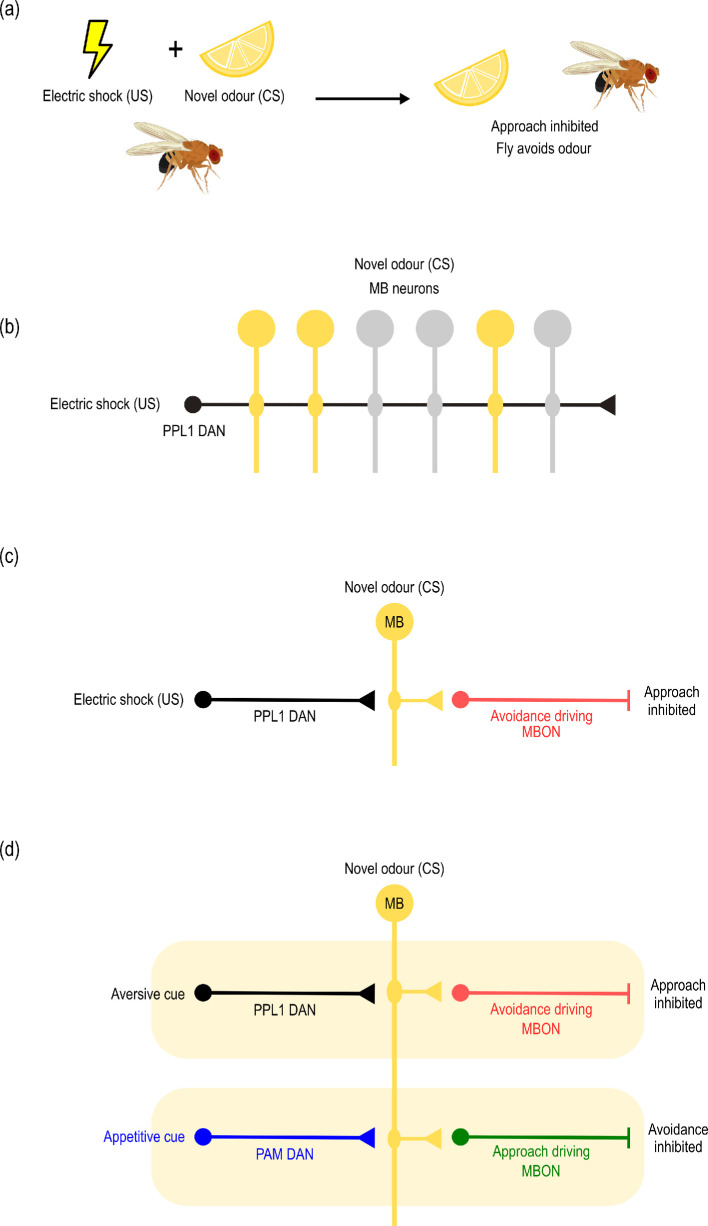
A model of aversive olfactory conditioning in *D. melanogaster.* (a) Simultaneous or temporally overlapping exposure to an odour (conditioned stimulus; CS) and electric shock (unconditioned stimulus; US) results in learnt avoidance of that odour. (b) Exposure to the odour activates a unique subset of mushroom body neurons (MB neurons), which constitute the neural trace of the CS. Dopaminergic neurons (DANs) from the PPL1 cluster synapse into the MB neurons and respond to the negative valence of the US. (c) Coactivation of any MB neuron and an incoming PPL1 DAN leads to suppression of nearby synapses with mushroom body output neurons (MBONs). (d) The mushroom bodies are divided into discrete compartments, such that incoming negative-valence PPL1 DANs are close to approach-driving MBONs, and positive-valence PAM DANs are close to their avoidance driving equivalents (note that ’approach-driving’ and ’avoidance-driving’ are simplifications of a much more complex reality, for the purpose of illustration). Aversive input therefore suppresses only approach behaviour and appetitive input suppresses avoidance behaviour. Figure is based on model description in [9,53,54]

Memory begins with acquisition, or learning, where sensory input is carried to the brain and changes the physiological state of a small subset of neurons, altering their synaptic inputs and outputs [9]. To put this in real, physical terms, we can consider a well-established model of aversive conditioning in *D. melanogaster*, developed through an enormous body of research that aims to understand how flies learn to avoid a particular odour because it predicts something unpleasant, like electric shock (figure 2a).

Initially, when an untrained fly detects an odour, information about the pattern of activation of olfactory receptors is processed first in the antennal lobe, then relayed through projection neurons to the mushroom bodies. Upon arrival, this input activates a subset of mushroom body neurons (MB neurons; also called Kenyon cells), producing an activation pattern that is unique to that odour [55] and can be thought of as its neural representation (figure 2b). At the same time, detection of the electric shock activates a set of dopaminergic neurons (PPL1 DANs) that synapse into the MB neurons, including those that have been activated by the odour exposure (figure 2c; [56]). Thus, two sets of neurons are co-activated: the unique set of MB neurons representing the specific odour that was encountered (*conditioned stimulus*), and the negative-valence PPL1 DANs that send information about a negative stimulus (electric shock; the *unconditioned* stimulus) into them. It is this concurrent activity that is key to producing learning, because it activates cAMP signalling pathways that weaken outgoing synaptic connections from the MB neurons to nearby mushroom body output neurons (MBONs; figure 2c). In this case, those outgoing neurons control approach behaviour, passing the signal downstream to integrative circuits that action the output. As a result of this change in connections between the odour’s specific representation pattern and the output neurons, approach behaviour is reduced next time the fly encounters the same odour [9,53,55]. The fly has thus learnt to stay away from a specific odour that was previously considered neutral.

We can expand on this simple circuit description to explore how evolutionary changes in one behavioural context might (or might not) impact others. To start, we compare the process of aversive olfactory conditioning in *Drosophila* with appetitive learning (where the valence of the outcome is positive, such as a sugar reward) to the same odour. During appetitive learning, the odour is represented in the same way, through activation of a unique trace in the MB neurons, but positive outcomes like sweet taste activate a different cluster of dopaminergic neurons to aversive stimuli (PAM DANs, rather than PPL1 DANs) [57]. Just like the negative-valence PPL1 DANs, these positive-valence PAM DANs synapse into the MB neurons containing the odour trace, and their shared activation again serves to weaken nearby onward synaptic connections with the outgoing output neurons (MBONs; figure 2d). In other words, the input that represents the unconditioned stimulus comes via a different route, but still inputs into the population of MB neurons that have come to represent the odour. How, then, is a different response produced? This is achieved in a simple way that does not demand differences in biochemical signalling. In the fly, the positive-valence PAM DANs simply synapse into the MB neurons near the output neurons that relate to avoidance, rather than approach [53]. Negative-valence PPL1 DANs and their relevant approach-related MBONs are in a physically different compartment of the fly mushroom bodies, and the MB neurons run through both (figure 2d). So, when positive-valence (appetitive) inputs are activated, the nearby output neurons are avoidance-driving, and avoidance becomes suppressed. The fly learns to approach the odour [9,53,54].

This description simplifies what is already a minimally adequate model of a complex process, and ignores the role of another neural structure—the lateral protocerebrum (LP), which is connected to both the mushroom bodies and the antennal lobe—in insect olfactory learning [58]. However, it serves to illustrate the point that the neural circuitry underlying olfactory learning can be evolutionarily modified in one context without affecting another. For example, although food and water can both be positive experiences for hungry/thirsty flies, it might be useful to learn about them differently. Accordingly, the two stimulus types elicit activity in different specialized neurons within the generally positive PAM DAN cluster [55,59]. Even within the category of ‘food’, a subpopulation of PAM DANs respond to sweet taste and real nutritious content, respectively, and only the latter leads to the formation of long-term memories [60]. Since the PAM DAN cluster responds to stimuli of positive valence, this increasing refinement of the appetitive conditioning process should have little effect on aversive conditioning. On a wider scale, there is increasing evidence of multiple pathways connecting to the basic learning circuit described above, which modulate its action according to the organism’s internal state (e.g. satiation, stress) [61–64], producing independent context-specific modifications. By contrast, modifications to the odour template captured by the MB neurons might affect both appetitive and aversive processes, since the odour representation is common to both. For example, animals with fewer inputs to each MB neuron cell, which will thus respond to a narrower range of odours, are better at discrimination tasks [65], and this improved discrimination ability would be expected to apply to both aversive and appetitive tasks.

As we alluded to above, odour learning also occurs to a more limited extent in a second neural pathway that bypasses the mushroom bodies entirely, proceeding from the antennal lobes to the LP. This pathway is associated with innate odour responses, but these responses can be modified by learning so that new valence is assigned to an odour here [58]. This contribution of multiple neural circuits to the same behavioural output is a key feature of memory systems in general [66] and it is important because it may allow evolutionary changes in one context to be achieved without complete loss of the original function [66], relaxing a hurdle that could otherwise constrain natural selection. The development of new functions through degeneracy and recycling is the focus of neural re-use theory, which highlights that the brain-wide networks underlying particular tasks are not unique to those tasks, and alternative realignments can produce additional new functions [48,67]. Following this view, later-evolving uses could recycle or co-opt pre-existing circuitry in disparate areas of the brain, such that highly derived behaviour could be achieved simply through different rearrangements of the same pre-existing infrastructure, which remains available for the original function. Overall, the available evidence thus suggests that fine-tuning in one context is certainly achievable without compromising or affecting others.

## 4. Short-term memory processes

STM processes are common to vertebrates and invertebrates, can be elicited by a single exposure event, and do not involve protein synthesis, relying instead on modification of existing proteins [68,69]. The question of whether STM is simply a precursor of LTM, or a separate independent storage process, was resolved in the late 1990s through pharmacological treatments that suppress STM formation without impacting LTM in rodents, producing rats that forget to avoid an electric shock grid when tested within hours of training, but nonetheless remember the next day [6,7]. Later work involving suppression of activity in different neuronal populations demonstrated the same phenomenon in *D. melanogaster* [5]. In addition to being separate to LTM, STM is also physiologically and conceptually distinct from working memory, which is a non-archival process that allows for active management of events that have been either recently experienced or recalled [6,70,71], and simply lingers until electrical activity and/or gas-based rapid signalling fades [7,72]. By contrast, STM is an archival storage mechanism that is maintained for a relatively short time, becoming inaccessible within minutes to hours, depending on the species and context [8,73].

Given that STM is distinct from other forms of memory, could it evolve to fulfil specific ecological tasks that require short-term retention? Potentially, although the importance of different temporal forms of memory within real ecological niches has received little empirical attention. However, for foraging bees, STM of individual flower visits has been hypothesized to allow individuals to keep track of reward levels within flower patches and decide whether to leave the patch or stay, without necessarily invoking LTM, which may be more relevant to decisions about which patch to return to [73–75]. Performance in a laboratory task that requires memories to be retained on the timescale of insect STM, and then forgotten, is a predictor of foraging efficiency in the wild for bumblebees [76], although only in flower-rich environments, and it remains to be seen whether this effect is specifically driven by STM. Otherwise, there are relatively few studies that relate any form of memory performance to real-world ecological performance, and those that do typically focus on tasks that require longer-term retention [26,77–80]. A first step would be to explore the extent to which selection on STM storage capacity affects LTM performance, which is a difficult task to achieve in natural environments but potentially approachable through experimental evolution in the laboratory, as we will return to below.

Memories stored in the STM may be useful, but they are also rapidly lost. This forgetting is not simply a natural decay process, but also an active process that is constantly promoted by the brain from acquisition onwards [34]. Active forgetting occurs through a range of mechanisms, the best-known of which involves the *Rac* gene family and protein products, which influence the size and shape of synaptic spines and thus alter synaptic connectivity [81]. In a seminal study, Shuai and colleagues showed that mutant flies lacking normal *Rac* activity acquired short-term memories as normal after exposure to a single olfactory conditioning trial but remembered them for more than one day [82], when wild-type flies would normally lose such single-trial associations within a few hours. Elevated *Rac* expression leads to exceptionally rapid forgetting [34]. Further work has shown that the activation of *Rac* to initiate synaptic remodelling is triggered by specialized ‘forgetting cells’, which are a third cluster of dopaminergic neurons that synapse into MB neurons [83], and that this pathway is also influenced by a number of other memory suppressor genes [11,84] such as *Scribble*, which codes for a scaffolding protein that physically interacts with *Rac* [85]. Evidence has also emerged for conserved *Rac-*induced forgetting in vertebrates, albeit more context-specific than in *Drosophila* [86].

On an ultimate level, why delete these short-term memories, rather than letting them fade? Intuitively, forgetting seems necessary to allow room for new storage without mammoth indexing requirements, but the fact that *Rac* is also implicated in reversal learning highlights that forgetting is also important for updating information. Flies that are exposed to two contrasting associations (e.g. odour predicts shock, then odour predicts no shock) typically develop a stronger memory for the most recent experience, but *Rac-*deficient flies have limited ability to over-write the original memory [82]. Is this an example of limited behavioural flexibility, as is sometimes suggested? Possibly, but extremely rapid reversal seems unlikely to be adaptive in many contexts. For example, a foraging bee would do better to judge the rewards offered by a flower patch by averaging over several flowers visited in succession, rather than simply leaving on encountering an empty flower, and accordingly, successful reversal learning usually requires more trials than initial learning. Hence, reversal learning is another trait that does not exist on a scale of ‘good’ or ‘bad’, but that could be matched to ecological tasks by natural selection.

A second interesting function of *Rac* is its contribution to trace conditioning, in which predictive stimuli (e.g. odour) and their unconditioned counterparts (e.g. shock) are separated in time. Trace conditioning requires neurons that respond to the conditioned stimulus to prolong their activity into the trace interval, requiring extra neural circuitry in *Drosophila* odour conditioning [87] and the contribution of additional neural regions to ‘normal’ (delay) conditioning, where conditioned and unconditioned stimulus exposures overlap temporally in vertebrates [88]. Consistent with *Rac*’s contribution to deletion of short-term memories, flies with deficient *Rac* function associatively link stimuli that are more distant in time than wild-type flies, which fail to associate all but near-successive events [87]. This might be useful when biologically important associations are temporally distinct, such as predation contexts where sound or odour cues predict the presence of an as-yet unseen predator. Moreover, while delays in trace-conditioning assays are usually in the region of seconds, the psychological literature includes the famous Garcia effect, whereby rodents learn to associate taste stimuli with nausea that occurs hours later [89]. While the major differences in timescale mean that the underlying machinery is likely to be distinct from that responsible for trace conditioning, these effects highlight that changes to the temporal continuity between stimuli required for learning thus represent another potential axis of variation that could be shaped to fit particular ecological tasks.

Active forgetting provides a means to remove short-term memories, but what about longer-term ones? Interestingly, molecular pathways required for forgetting are not the same for all forms of memory; thus, forgetting probably evolved through the action of selection on multiple targets, such that changes relating to one memory phase need not be consistent across others. For example, *Rac* is not required for forgetting of ARM, which instead requires the protein Cdc42, derived from the same family [90,91]. Other gene products and/or receptor types may perform a similar function for LTM in rodents [92], but in many cases it is difficult to tell whether effects reflect active removal of LTM or simply lack of consolidation of the memory in the first place [93], a process that we will discuss in more detail in the next section.

## 5. Longer-term memory

Classic, evolutionarily conserved LTM is a process that requires novel protein synthesis (but see [94]), producing memories that guide behaviour in the region of days to months and—in some species—beyond. However, it is not the only form of storage that occurs in the longer-term. In *Drosophila* and most likely also other insects [95], ARM also exists, lasting for less time but without requiring protein synthesis, and typically forming after a single exposure event [8]. The question of whether memories of the same experience can sit in both ARM and LTM attracts debate [8], but mutant flies that are unable to form LTM—which would normally accrue during spaced training—remember *less* the more training they receive [4]. Presumably, the memory in ARM is being actively removed by spaced training that would normally elicit LTM, but the modified flies have built no LTM to replace it with. This implies that the two are alternatives, and formation of memories in LTM causes active deletion from ARM.

LTM engrams can last longer than their ARM equivalents because protein synthesis contributes to stabilizing neural networks through physical changes in the strength and/or number of connections [9]. For example, storage of long-term olfactory memories relies on active remodelling of synaptic microcircuits within the mushroom bodies of honeybees, fruit flies and leaf-cutting ants [37,40,96]. This process is interesting from an evolutionary point of view because there is considerable evidence that it is energetically demanding. For example, mutant LTM-deficient flies remember an odour/shock association 24 h after exposure when starved throughout, but not when fed, but wild-type flies with intact LTM remember under both conditions [52], implying that starved flies selectively shut down LTM but not ARM. Forcing starved wild-type flies to form LTM—which is achieved by upregulating mushroom body energy flux—is associated with increased mortality [52], suggesting a draw from energy stores that support other fitness-critical processes. Even under more mild nutritional stress than starvation, repeated exposure to conditioning has been shown to impact egg-laying rate and/or mortality in flies selected for high learning rates [97] and in honeybees [98], although the same was not true in a more naturalistic context for bumblebee queens [99]. Finally, flies double their sucrose intake after exposure to stimulus pairings that lead to LTM formation [52]. This effect also occurs in an appetitive context in bees, albeit on a longer timescale [100]. Note that the costs of LTM formation may not generalize to its maintenance, since behavioural expression of LTM in leaf-cutting ants outlives the increases in synaptic bouton density that accompany its formation, returning to baseline after an initial increase [96]. This suggests a homeostatic process where strengthening active connections is compensated by eliminating other ones, hence allowing the consolidation of new memories without saturating brain memory capacity.

LTM formation is energetically costly, but ARM is more than simply a cheaper alternative: it functions as a store for those memories for which future reliability is uncertain. The criteria for entry to LTM, and therefore to guide behaviour well into the future, are narrow, typically (but not universally) involving multiple repeated co-occurrences which must be separated by time delays [8,14]. As a result, associations must reach a higher consistency criterion for entry to LTM, and interestingly, this criterion can vary between stimulus types: for example, memories of ‘dangerous’ stimuli enter quickly but memories of ‘safe’ stimuli require more exposure and repetition [101]. For a long time, the mechanisms by which this spaced repetition-based criterion for entry could be achieved remained unclear, but computational modelling approaches have highlighted how simple interplay between dual or multiple signalling cascades could achieve such effects [102]. For example, in the sea slug *Aplysia,* exposure to a stimulus elicits two different biochemical cascades that operate on different timescales. One cascade is slow, taking 45 min to peak, while the other is rapid and transient, returning to baseline levels within 15 min. These differing timescales mean that peaks are initially out of phase but eventually occur simultaneously after multiple exposures, potentially eliciting an additive or synergistic response that is necessary for LTM formation [103]. In this way, LTM storage of co-incidental associations that have little value in predicting future rewards could be minimized.

Whether this *Aplysia* model and/or other signalling cascades or circuit-based alternatives in other species [104,105] prove to underlie the mysterious and taxonomically general spacing requirement for LTM formation remains to be seen [9], and the fact that LTM has now been shown to sometimes form after a single trial [94,106] suggests that the role of signalling cascades may not be universal. However, it is achieved, though the gating of LTM is interesting from an evolutionary point of view because natural selection may be able to fine-tune the criteria according to the likely reliability of a predictive cue or the reward value. For example, the wasp *Cotesia glomerata* is a parasitoid of the cabbage white butterfly *Pieris rapae*, which typically lays single eggs on a variety of plants. Since being parasitized is very much against the interests of the host caterpillar, the butterfly strategically lays single eggs on different plants in the vicinity, so that learning to focus on one single plant type where an egg has been found may not always be optimal. Accordingly, the wasp does not form protein synthesis-dependent LTM of the plant type upon which it finds an egg until the association has occurred multiple times [107]. However, when parasitizing caterpillars of a closely related butterfly *Pieris brassicae*, the same association is gated differently, such that LTM forms immediately on a single exposure. This is congruent with differences in the behaviour and value of the hosts, since *P. brassicae* lays clusters of eggs on the same plant. Hence, the association between plant type and a more profitable reward is reliably valuable in the future.

This pattern is not unique to *C. glomerata*, but is replicated in a taxonomically distant wasp that parasitizes the eggs of the same two butterfly species [16]. Indeed, variation in the readiness to form LTM exists across a variety of species and scenarios [17,20,108], including in *D. melanogaster*, where a natural polymorphism in the *for* gene that drives sedentary or dispersal-based foraging also affects memory, such that sedentary flies (‘sitters’; *for*^S^ variant) perform better in olfactory learning tasks that require LTM [109]. Memories formed by ‘rovers’ (*for*^R^ variant) are instead biased to recent events, and are subject to retroactive interference, whereby recent memories displace older ones [110]. The readiness with which memories can be lost or suppressed through extinction, which occurs when a conditioned stimulus (e.g. flower colour, plant volatile) that was previously rewarding (e.g. predicted nectar, caterpillar presence) is repeatedly encountered in the absence of reward, is also a potential source of diversity, as is the extent to which such associations can be spontaneously recovered in new contexts [111,112].

## 6. Summary and perspectives

The perspective offered here has been that the neuroscience literature could be a source of inspiration for those interested in exploring the diversity of memory, because it uncovers axes along which natural selection could act. We have suggested that such axes might include variation in forgetting, in STM capacity, in preparedness to form long-term memories, and in the temporal gaps between stimuli needed for learning, but these are simply a few examples. Researchers interested in the evolution of cognition across taxa have historically looked to psychology for explanations of mechanism, and it would be correct to say that each of these ideas could equally well come from that field, particularly from the exceptionally rich literature on the psychology of associative learning [113]. However, what neuroscience adds is critically important: an idea of the adaptive landscape to clarify what changes to expect; tools to detect those changes experimentally; and most importantly, evidence to predict what might change alongside.

Could we systematically map variation in the traits that we have discussed above to ecology across multiple species? If we had no guidance as to what species to target for behavioural assays, quite possibly not. Taxonomically-wide phylogenetic analyses that attempt to link cognitive variation to specific ecological parameters (e.g. primate frugivory) lack information about fine-scale diversity in memory parameters that require careful measurement, and must instead rely on broad-brush but relatively easily comparable traits such as relative or absolute brain size [114]. A comparative behavioural approach might theoretically be possible across *Drosophila* species, since about 90 of these can be easily bought commercially and could be tested in standardized, quick laboratory procedures—but for most of them, we know little about their ecology and so mapping cognitive variation to ecology would be difficult. However, if we have strong hypotheses about specific species to target, based on marked differences in ecology from closely related sister groups, this endeavour becomes much easier. Other authors have successfully applied this targeted approach to specific species to provide evidence that particular aspects of cognition have responded to selection. For example, *Heliconius* butterflies that specialize on pollen-feeding from a particular host plant have been shown to differ in their LTM formation in comparison to their exclusively nectar-feeding, less specialist close relatives [115]; the genome of a *Polistes* paper wasp species that relies on facial patterns for individual recognition shows evidence of selection on learning and memory [116] and *Poecile* chickadee species that rely heavily on food caching during high-altitude winters show evidence of hippocampal expansion relative to those that do not [27]. In each of these cases, the finer details of how memory has changed on a mechanistic level, relative to comparator species, are not available—but it is for a similar body of work on parasitoid wasps, as described above [16,20,107,108,117]. We suggest that such targeted approaches could easily be applied to many insects where interesting variation in ecology occurs within a species group. Examples might be *Ammophila* digger wasps, where some species are progressive provisioners that must remember the locations of specific nests [118]; *Bombus* (bumblebees), where some species are broad generalists and others highly specialist [119], or *Polistes* paper wasps, where socially parasitic species must usurp queens of other species to take over the nest [120], but these are just a few examples from our own specialist area, and there will be many, many more.

An alternative approach that we think is exciting is to allow evolution to run in the laboratory, manipulating environments in a manner that is expected to affect the benefits of learning and memory [97,121–124]—a technique that is well-suited to insect laboratory models with short generation times. For example, manipulating the reliability of the association between odour cues and suitable egg-laying substrates influences the tendency to form medium/long-term olfactory memories, while changing the predictive power of particular cue types (e.g. odour versus colour) alters the tendency to learn about them [124,125]. Although experimental evolution does not capture selection over millennia, and thus may not necessarily be an accurate representation of endpoints in the real world, it would certainly be informative to explore exactly *what* changes in these paradigms to generate hypotheses for comparative work (e.g. [117]).

Most of the studies discussed above relate to *D. melanogaster*, because the development of neurogenetic tools for manipulation of memory has been focussed on here. Although many aspects of memory are deeply evolutionarily conserved, the structure and circuitry of other insect brains will naturally be the closest match in terms of generalities of mechanisms. This is not a limitation, because an insect focus is not taxonomically narrow, or specialist: the majority of animal species on the planet are insects [29,30]. Beetles alone constitute about 25% of the world’s described animal species [29], but their cognitive abilities are almost unexplored. The animal world’s ecological diversity is represented in the insects: from eusocial to solitary, necrophages to phytophages, parasitoids to hosts, hunters to prey, caring parents to species that die long before their offspring are born. They capture variation in ecological selection pressures like no other group, and although they will never be a good focus for understanding those cognitive traits that are limited to humans and their close relatives, those are far from the only cognitive traits worth studying. The basic ability to acquire, consolidate, recall and forget memories is fundamental to cognition, and neuroscience-informed ecology is already proving useful in exploring the directions in which evolution can take these traits.

## Data Availability

This article has no additional data.
